# Non‐ICANS neurotoxicity after BCMA‐directed CAR‐T therapy: Clinical spectrum, outcomes, and a framework for neurology–oncology co‐management

**DOI:** 10.1002/hem3.70404

**Published:** 2026-06-15

**Authors:** Jan‐Michael Werner, Philipp Gödel, Norbert Galldiks, Christopher Krone, Michael Schroeter, Udo Holtick, Ruth Flümann, Gilbert Wunderlich, Michael Hallek, Gereon R. Fink, Christoph Scheid, Oezguer A. Onur, Tim Richardson

**Affiliations:** ^1^ Department of Neurology, Faculty of Medicine and University Hospital Cologne University of Cologne Cologne Germany; ^2^ Department I of Internal Medicine, Faculty of Medicine and University Hospital Cologne University of Cologne Cologne Germany; ^3^ Institute of Neuroscience and Medicine (INM‐3, INM‐4) Research Center Juelich Juelich Germany; ^4^ Center for Rare Diseases, Faculty of Medicine and University Hospital Cologne University of Cologne Cologne Germany

Ciltacabtagene autoleucel (cilta‐cel), a B‐cell maturation antigen (BCMA)–directed chimeric antigen receptor T‐cell (CAR‐T) therapy, has demonstrated deep and durable responses in patients with relapsed or refractory multiple myeloma and is rapidly moving into routine clinical practice worldwide.^1^, ^2^ While cytokine release syndrome (CRS) and immune‐effector cell‐associated neurotoxicity syndrome (ICANS) are well described, increasing attention is directed toward rarer but potentially severe neurotoxicity outside the ICANS spectrum.^3^, ^4^ Non‐ICANS immune‐effector cell‐associated (IEC) toxicities reported after BCMA‐directed CAR‐T therapies include movement/neurocognitive treatment‐emergent events with Parkinsonism‐like features (IEC‐PKS), cranial nerve palsies (IEC‐NP), Guillain–Barré‐syndrome‐like presentations (IEC‐GBS), and myelopathy.^5^ Phenotypes can overlap and evolve over weeks, complicating recognition and therapeutic decision‐making.

The biological basis of delayed non‐ICANS neurotoxicity after BCMA‐directed CAR‐T therapy is an active area of investigation. Early reports have detected CAR‐T in the cerebrospinal fluid (CSF) during neurotoxicity and, in individual cases, have described neuropathologic evidence of lymphocytic infiltration in affected brain regions, findings compatible with central nervous system (CNS) trafficking and the persistence of CAR‐T, together with local inflammatory tissue injury.^4^ These observations have raised the possibility of an on‐target/off‐tumor mechanism in susceptible neural tissues, although causality has not been established.^4^, ^6^ At the cohort level, delayed non‐ICANS neurotoxicity has been linked to pronounced CAR‐T expansion and persistence and to heightened immune activation, often in patients with clinically relevant CRS and/or ICANS, suggesting that both cellular kinetics and inflammatory activation may contribute.^3^, ^5^, ^6^, ^7^, ^8^ Despite these signals in clinical trials and real‐world settings, non‐ICANS neurotoxicity remains insufficiently characterized, and data from large in‐label cohorts are limited. Against this background, this report pursues two aims: (1) to characterize the clinical spectrum, management, and outcomes of severe non‐ICANS IEC neurotoxicity in a real‐world in‐label cilta‐cel cohort, and (2) to propose a structured framework for triage, diagnostic evaluation, and initial neurology–oncology co‐management applicable beyond specialized CAR‐T centers.

We conducted a retrospective single‐center analysis within a predefined hematology–neurology collaboration. Consecutive patients receiving in‐label cilta‐cel for relapsed or refractory multiple myeloma between May 2023 and December 2025 were included (*n* = 117; median age, 69 years [range, 59–79]; median follow‐up, 11.3 months [range, 1–31.5]). Severe non‐ICANS neurotoxicity was distinguished from ICANS per EBMT recommendations^6^ and adjudicated by joint hematology–neurology review. Cases of severe non‐ICANS neurotoxicity were identified after exclusion of alternative causes (infection, metabolic/toxic disturbances, medication effects, and CNS myeloma/relapse).

Ten patients (8.5%) met the criteria, with the majority primarily treated on oncology wards with structured inpatient neurology consultation and outpatient follow‐up. Patient characteristics are summarized in Table 1; timing, overlap, management, and outcomes are shown in Figure 1. Non‐ICANS IEC neurotoxicity began a median of 45 days post CAR‐T infusion (range, 11–160 days). IEC‐GBS occurred in three patients (all CTCAE Grade 5; Days 24–97) and followed fulminant courses despite escalation from IVIG and corticosteroids to intensified immunosuppression and extracorporeal antibody/cytokine depletion. All three patients deceased 3–7 weeks after the diagnosis of IEC‐GBS. Median time from symptom onset to high‐dose corticosteroids was 3.5 days (range, 2–13); the start of corticosteroid treatment for each patient is depicted in Figure 1A. Notably, immune‐directed escalation coincided with marked declines in circulating CAR‐T copies, yet without neurologic recovery. IEC‐PKS developed in three patients (CTCAE Grades 2–4; Days 45–160) and was persistently dopamine‐nonresponsive. Two patients underwent intensified immunosuppression (including chemotherapy) and deceased from infectious complications in the context of cytopenia; one remains alive with ongoing symptoms. Five patients had severe IEC‐NP, predominantly peripheral facial palsies. One case included oculomotor palsy as part of multisyndromic toxicity. Treatment was generally corticosteroid‐based, with escalation in multisyndromic neurotoxicity. Improvement occurred in some cases after intensification of immunosuppression, whereas deficits persisted in a patient with facial palsy and concomitant IEC‐encephalitis despite high‐dose corticosteroids plus etoposide and intrathecal chemotherapy. Two IEC‐NP cases occurred within broader syndromes of IEC‐PKS or IEC‐GBS. IEC‐encephalitis was observed in two patients (CTCAE Grade 4; Days 11 and 39). Both received high‐dose corticosteroids (with additional etoposide and intrathecal chemotherapy in one); encephalitis resolved in one patient (while concomitant IEC‐PKS persisted), whereas encephalitis persisted in the other at the last follow‐up.

**Table 1:** Clinical and treatment characteristics of multiple myeloma patients treated with B‐cell maturation antigen (BCMA)–directed chimeric antigen receptor T cell (CAR‐T) and non‐immune effector cell‐associated neurotoxicity syndrome (non‐ICANS) immune‐effector cell‐associated (IEC)‐neurotoxicity.

Characteristic	All patients (*n* = 10)
*1. Patient demographics and clinical background*	
Age at infusion, years (median)	69 (range, 59–79)
Sex (M:F ratio)	3:2
ECOG PS at CAR‐T infusion (median)	1 (range, 0–1)
R‐ISS stage at diagnosis (median)	I (range, I–III)
High‐risk cytogenetics (IMWG 2025)	50% (*n* = 5)
Prior lines of therapy (median)	3 (range, 2–5)
Triple‐ or penta‐refractory disease	70% (*n* = 7)
Extramedullary disease	30% (*n* = 3)
Prior BCMA‐directed therapy	None
Absolute CD3^+^ cells at apheresis, ×10^9^/L (median)	2.77 (range, 1.01–6.81)
Absolute lymphocytes at apheresis, ×10^9^/L (median)	0.71 (range, 0.22–1.50)
Response to bridging therapy	
Complete response	30% (*n* = 3)
Partial response	30% (*n* = 3)
Stable disease	20% (*n* = 2)
Progressive disease	20% (*n* = 2)
Vein‐to‐vein time, days (median)	78 (range, 56–89)
Baseline creatinine at CAR‐T, mg/dL (median)	0.78 (range, 0.47–2.97)
Baseline albumin at CAR‐T, g/L (median)	35.5 (range, 29–44)
*2. CRS and ICANS characteristics and management*	
CRS occurrence	90% (*n* = 9)
CRS Grade ≥3	10% (*n* = 1)
Duration of CRS, days (median)	2 (range, 1–4)
ICANS occurrence	20% (*n* = 2)
ICANS Grade ≥3	None
Duration of ICANS, days (median)	6 (range, 4–8)
Use of tocilizumab	90% (*n* = 9)
Use of dexamethasone for CRS/ICANS	30% (*n* = 3)
Best overall response	CR 100% (*n* = 10)
*3. Treatment of non‐ICANS IEC neurotoxicity*	
Corticosteroids (50–200 mg dose equivalent prednisone)	100% (*n* = 10)
‐ Duration of corticosteroid treatment, days (median)	13 (range, 4–32)
Intravenous immunoglobulin (total 2 g/kg)	30% (*n* = 3)
Etoposide (150–300 mg/m^2^)	30% (*n* = 3)
Cyclophosphamide (1–4 g/m^2^)	30% (*n* = 3)
l‐Dopa	30% (*n* = 3)
Dasatinib 100 mg/day	20% (*n* = 2)
Immunoadsorption	20% (*n* = 2)
Intrathecal chemotherapy (15 mg methotrexate, 40 mg cytarabine, 4 mg dexamethasone)	20% (*n* = 2)
Intravenous anakinra 8 mg/kg/day	10% (*n* = 1)

Abbreviations: CR, complete response; CRS, cytokine release syndrome; ECOG PS, Eastern Cooperative Oncology Group performance status; IMWG, International Myeloma Working Group; R‐ISS, Revised International Staging System.

**Figure 1:**
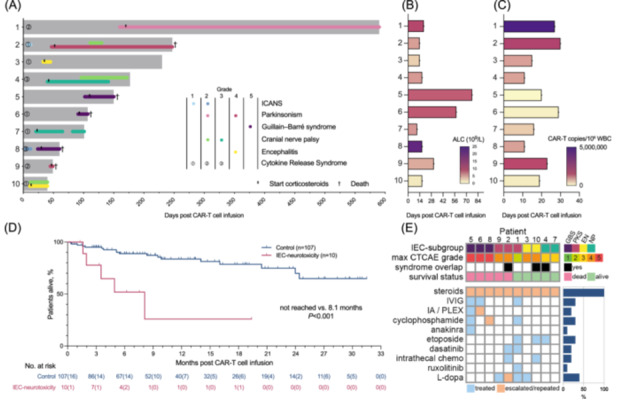
**(A) Swimmer plot of the 10 patients with immune‐effector cell‐associated (IEC)‐neurotoxicity following chimeric antigen receptor T‐cell (CAR‐T) treatment for multiple myeloma.** Each row represents one patient, sorted by overall survival after CAR‐T infusion. The time to non‐immune effector cell‐associated neurotoxicity syndrome (non‐ICANS) neurotoxicity ranged from 2 to 23 weeks. The grades of ICANS, neurotoxicity, and cytokine release syndrome are color‐coded. **(B)** Time to peak of the absolute lymphocyte count (ALC) for each patient with a color scale visualizing the individual peak ALC values (range, 1.0–22.2 × 10^9^/L). **(C)** Time to peak CAR‐T copies in peripheral blood samples after CAR‐T infusion, assessed via polymerase chain reaction (PCR): the color scale visualizes the individual peak CAR‐T copy count (range, 39,553–4,970,000 copies/10^6^ leukocytes). **(D)** Kaplan–Meier curves for the overall survival in patients with IEC‐neurotoxicity and in those without (control). Numbers at risk are shown at specified time points; numbers in parentheses indicate censored observations within the subsequent interval. **(E)** Treatment and outcome oncoprint of IEC‐neurotoxicity. Columns represent individual patients, top annotation tracks indicate IEC neurotoxicity subgroup, maximum CTCAE grade, syndrome overlap, and survival status. The matrix displays therapies administered for IEC‐neurotoxicity; blue denotes treatment given, and orange denotes escalation/repeated administration (white, not given). Right‐hand bars show the proportion of patients receiving each therapy (%).

IEC‐neurotoxicities were frequently life‐threatening (CTCAE Grades 4 and 5), and fatal outcomes often occurred in the setting of prolonged immunosuppression with severe infections. Syndromes overlapped or evolved sequentially, prompting stepwise escalation to broader anti‐inflammatory, cytotoxic, and/or CSF‐directed approaches. In several cases, CAR‐T copy numbers decreased in peripheral blood and/or CSF after systemic or intrathecal chemotherapy, yet neurologic trajectories remained poor or static. Symptomatic dopaminergic therapy was commonly ineffective.

Before CAR‐T infusion, 6 of the 10 neurotoxicity patients had at least a partial response to bridging therapy; 2 had stable, and 2 had progressive disease. Two patients developed ICANS (Grades 1 and 2) plus non‐ICANS neurotoxicity (Table 1). Following cilta‐cel, all 10 achieved a complete response, and none have progressed. Non‐relapse mortality among neurotoxicity patients was 50% (three deaths attributed to direct neurotoxicity and two to infectious complications after immunosuppression), compared with 8.4% among patients without IEC‐neurotoxicity (including two patients dying after IEC‐enterocolitis). Patients with non‐ICANS IEC‐neurotoxicity had markedly inferior survival (median survival 8.1 months vs. not reached in controls; log‐rank, P < 0.001) (Figure 1D). At the last follow‐up, five patients with neurotoxicity were alive, including three patients with persistent neurologic deficits and two patients with partial or ongoing neurologic improvement under continued supportive or immune‐directed therapy. Taken together, the combination of delayed onset, frequent overlap of syndromes, and high non‐relapse mortality, often compounded by infectious complications during immunosuppressive escalation, argues for standardized co‐management beyond the early ICANS window. These observations form the empirical basis for the following clinical framework, which we propose as a practical guide for hematology–neurology co‐management applicable beyond specialized CAR‐T centers.

No evidence‐based treatment guidelines currently exist for non‐ICANS IEC neurotoxicity, and management remains largely empiric, highlighting the urgent need for prospective registries and standardized treatment algorithms. We therefore advocate protocolized neurology–oncology co‐management for any new neurologic symptom after CAR‐T infusion (focal deficit, cranial neuropathy, new movement disorder, progressive weakness, encephalopathy, and seizures).

A simple three‐level triage can standardize urgency across teams:

(1) emergent‐rapid progression, bulbar/respiratory compromise, severe encephalopathy, or refractory seizures require intensive care unit (ICU) monitoring and immediate neurology input
(2) urgent, new disabling deficits without imminent airway/hemodynamic threat should be reviewed by neurology within 24 h
(3) suspicious but stable symptoms should trigger a structured outpatient/inpatient work‐up within a few days with explicit return precautions.

Such shared language is particularly valuable when patients present to non‐CAR‐T centers.

Diagnostics should be protocolized rather than ad hoc. A core bundle includes targeted labs to exclude metabolic/toxic factors, magnetic resonance imaging (MRI) brain without and with contrast enhancement (and spine when myelopathy or radiculopathy is suspected), a spot electroencephalography (EEG) in patients with encephalopathy or seizures, with continuous EEG monitoring reserved for refractory seizures or suspected non‐convulsive status epilepticus, and CSF analysis (when safe and clinically indicated) including infectious testing, CAR‐T polymerase chain reaction (PCR), and cytokine/chemokine profiling where available) when safe and clinically indicated. Suspected peripheral nerve involvement warrants early electromyography/nerve conduction studies, autonomic assessment when relevant, and respiratory monitoring (e.g., forced vital capacity). For mnestic complaints or cognitive decline, neuropsychological testing can quantify deficits, establish baselines for follow‐up, and support syndromic classification. We recommend a baseline neurological assessment before CAR‐T infusion to document pre‐existing deficits and provide a reference for subsequent monitoring. Structured intermittent neurological follow‐up during the post‐infusion surveillance period may facilitate earlier recognition of emerging symptoms before overt progression. In‐house neurology is critical because patients may present at any time, including delayed phases beyond routine monitoring windows. To facilitate practical implementation, we provide a supplementary framework summarizing triage, core diagnostics, and immediate initial management steps (Supporting Information S1: Figure 1). At our center, in addition to inpatient consultative neurology, we have now implemented dedicated ambulatory rapid‐access options for suspected IEC‐neurotoxicity to shorten diagnostic latency, enable shared treatment decisions, and shorten time to treatment. Although the retrospective design precludes formal assessment of the effect of neurology co‐management on outcomes, delayed recognition in patients initially presenting to peripheral hospitals highlights the potential value of early neurological involvement.

Signals from prior cohorts and our data suggest that expansion kinetics may help identify patients who warrant intensified neurologic surveillance. Across the CARTITUDE studies, movement/neurocognitive toxicities have been linked to a pattern that includes high CAR‐T expansion/persistence and markers of heightened inflammatory burden (often alongside clinically relevant CRS and/or ICANS), supporting the concept that cellular kinetics matter in non‐ICANS.^7^ In biomarker analyses, absolute lymphocyte count (ALC) correlates with CAR+ T‐cell counts, and an association between higher early ALC and subsequent IEC‐neurotoxicity has been shown, making ALC a possible surrogate for expansion kinetics in routine monitoring.^9^ Real‐world data suggest that an ALCpeak > 3 × 10^9^/L identifies patients at higher risk of delayed neurotoxicity, but prophylactic oral dexamethasone in high‐ALCpeak patients did not reduce delayed neurotoxicity, suggesting alternative mitigation strategies are needed.^10^ In our cohort, 9 of 10 neurotoxicity patients had an ALCpeak > 3 × 10^9^/L (median Day 21; range, 11–77). Median CAR‐T expansion at onset of neurotoxicity was 222,907 copies/10^6^ leukocytes (range, 124–1,089,302 copies/10^6^ leukocytes). Patient‐level data on ALCpeak and CAR‐T expansion are presented in Figure 1B,C. Compared to patients without neurotoxicity, those with neurotoxicity exhibited both higher and later peak CAR‐T expansion (peak copies/10^6^ leukocytes: median 710,766 [range, 39,553–1,800,000] vs. 131,077 [range, 49,259–2,318,275], Mann–Whitney *U* test P < 0.001; time to peak: median days post‐infusion, 22 [range, 14–160] vs. 14 [range, 14–442], Mann–Whitney *U* test P = 0.0015). In most affected patients, copy numbers were already declining at diagnosis of neurotoxicity, but two patients showed copy numbers at or exceeding their recorded peak, indicating persistent or still‐rising expansion at symptom onset. Inflammatory markers such as C‐reactive protein (CRP), ferritin, and lactate dehydrogenase may be trended longitudinally as part of the overall clinical assessment, but they are nonspecific, and there is currently no proven benefit for management of non‐ICANS IEC neurotoxicity. This underscores the importance of repeated neurological assessment and timely re‐evaluation of new or evolving symptoms. In contrast, CAR‐T PCR in peripheral blood and, when clinically indicated, CSF may offer more directly relevant diagnostic information on persistent or ongoing CAR‐T expansion. As the pathophysiology of non‐ICANS IEC neurotoxicity remains under active investigation, more informative inflammatory biomarkers may yet be identified. For descriptive context, mean and individual CRP and ferritin values in the neurotoxicity cohort are shown in Supporting Information S2: Figure 2.

Multiple groups are now focusing on modifiable risk factors and safety‐enhancing measures for cilta‐cel, including programmatic approaches to reduce delayed neurotoxicity and non‐relapse mortality, but the optimal strategy for individual patients is still evolving.^11^ Therapeutically, “immune‐directed escalation” remains empiric, and the evidence is largely limited to single cases. For steroid‐refractory non‐ICANS neurotoxicities, CAR‐T ablation with cyclophosphamide has been reported as a mitigation approach, yet when and in whom to deploy such interventions is not established.^12^, ^13^ Given heterogeneous predictors across studies, systematic surveillance is critical to enable timely recognition and intervention when neurologic syndromes emerge beyond the ICANS window.^8^, ^14^

Limitations of our study include its retrospective single‐center design, small subgroup sizes, and currently non‐standardized management confounded by severity, precluding causal inference. Short follow‐up and censoring limit the ability to compare survival.

In summary, severe non‐ICANS IEC‐neurotoxicity following in‐label cilta‐cel was clinically heterogeneous, frequently delayed, and associated with substantial non‐relapse mortality and enduring morbidity despite aggressive treatment. Importantly, the risk window extends well beyond Day 30, underscoring the need for prolonged vigilance during routine response monitoring and explicit patient education on “red‐flag” symptoms. Furthermore, evaluating phenotypes requires early neurological assessment, and current escalation approaches may diminish quantifiable CAR‐T persistence without improving neurological function. This highlights the necessity for prospective registries that employ standardized case definitions, biospecimen collection protocols, and pre‐defined treatment algorithms. Given that cilta‐cel is increasingly used beyond specialized CAR‐T centers, these findings support the development of standardized protocols that incorporate neurology and hematology, and specify diagnostic criteria and treatment escalation strategies. Lastly, specialized training for local neurologists and oncology teams is crucial to ensure quick identification, effective triage, and coordinated decision‐making.

## AUTHOR CONTRIBUTIONS

**Jan‐Michael Werner**: Conceptualization; investigation; writing—original draft; methodology; formal analysis; data curation; visualization. **Philipp Gödel**: Investigation; data curation. **Norbert Galldiks**: Supervision; conceptualization. **Christopher Krone**: Data curation; resources. **Michael Schroeter**: Supervision; methodology. **Udo Holtick**: Conceptualization; validation. **Ruth Flümann**: Data curation; writing—review and editing. **Gilbert Wunderlich**: Writing—review and editing; supervision. **Michael Hallek**: Supervision. **Gereon R. Fink**: Supervision. **Christoph Scheid**: Conceptualization; investigation; validation; writing—review and editing; project administration. **Oezguer A. Onur**: Supervision; validation; writing—review and editing. **Tim Richardson**: Conceptualization; investigation; writing—original draft; methodology; formal analysis; data curation.

## CONFLICT OF INTEREST STATEMENT

J.‐M.W.: Nothing to disclose. C.K.: Nothing to disclose. P.G.: Nothing to disclose. N.G.: Honoraria for lectures from Servier, for advisory board participation from Telix Pharmaceuticals and Servier, and for consultancy services from Telix Pharmaceuticals. M.S.: Received speaker's honoraria from Alexion, Argenx, BVDN, Datamed Cologne, FomF, Grifols, Merck, Novartis, Roche, Simon&Kucher, and UCB. U.H.: Advisory Boards for Oncopeptides, GSK, and J&J. R.F.: Travel grants by Avanzanite. G.W.: Honoraria for advisory board attendance, speaker's fees from Roche, Novartis, Alnylam, and AstraZeneca outside of the present work. M.H.: Nothing to disclose. G.R.F.: Serves as an editorial board member of NeuroImage: Clinical, Zeitschrift für Neuropsychologie, Zeitschrift für Kinder‐ und Jugendpsychiatrie und Psychotherapie, and Info Neurologie & Psychiatrie; receives royalties for the books Funktionelle MRT in Psychiatrie und Neurologie, Neurologische Differentialdiagnose, SOP Neurologie, and Therapiehandbuch Neurologie; receives royalties for the neuropsychological tests KAS, NP‐KiSS, and KöpSS; receives honoraria for speaking engagements from the Deutsche Gesellschaft für Neurologie (DGN) and Forum für medizinische Fortbildung FomF GmbH. C.S.: Advisory boards for and received honoraria from Amgen, AbbVie, BMS, Janssen, Novartis, Oncopeptides, Pfizer, Roche, Sanofi, Stemline Menarini, and Takeda; has received research support from Janssen and Takeda. O.A.O.: Honoraria for Advisory Boards and talks for Eisai, Lilly, Roche, and GE. T.R.: Advisory Boards for Johnson & Johnson, Kite Pharma, Takeda, Amgen, and Sanofi.

## ETHICS STATEMENT

All patients provided written informed consent. The study was conducted in accordance with the Declaration of Helsinki and approved by the local ethics committee (no. 24‐1201‐retro).

## FUNDING

Open Access funding enabled and organized by Projekt DEAL.

## Supporting information

Supporting Information.

Supporting Information.

## Data Availability

The data that support the findings of this study are available from the corresponding author upon reasonable request.
