# Supplementary material for: Non‐ICANS neurotoxicity after BCMA‐directed CAR‐T therapy: Clinical spectrum, outcomes, and a framework for neurology–oncology co‐management
Source: Hemasphere. 2026 Jun 15;10(6):e70404. doi: 10.1002/hem3.70404 (PMC13266575; doi:10.1002/hem3.70404)
Supplement: Supplementary file 1 — Supporting Information. [file HEM3-10-e70404-s002.pdf]

New neurological symptom after CAR-T cell infusion

TRIAGE

Emergent

Rapid progression · bulbar/respiratory compromise
refractory seizures · severe encephalopathy

↓

ICU monitoring + immediate neurological consultation
In case of refractory seizures consider EEG monitoring and / or neurological intensive care

Urgent

New disabling neurological deficit and / or self-terminating seizures
without airway or hemodynamic instability

↓

Neurology review within 24 hours

Suspicious but stable

Subtle or non-progressive symptoms

↓

Structured diagnostic workup within 3 days + return precautions

Notify CAR-T center if patient presents at peripheral hospital

DIAGNOSTICS & INITIAL STEPS

Neurological evaluation

Define the neurological syndrome and assess clinical severity

\+

Laboratory

Metabolic/toxic exclusion · infectious workup · CRP, ferritin, LDH
absolute lymphocyte count · CAR-T cell detection in peripheral blood

\+

Imaging

MRI brain +/- contrast enhancement (+ spine in case of myelopathy/radiculopathy)
DaTSCAN / $[^{18}F]$FDOPA PET (in case of Parkinson syndrome)

\+

Neurophysiology

EEG · electromyography · nerve conduction studies depending on the clinical syndrome · forced vital capacity monitoring (in suspected peripheral nerve involvement)

\+

CSF analysis

Infection panel · CAR-T cell detection · cytokine/chemokine profiling (if available)

↓

Initial management

Start high-dose dexamethasone immediately (do not delay for complete workup)

↓

Joint neurology-oncology treatment management decision
