# Supplementary figures and images for: Non‐ICANS neurotoxicity after BCMA‐directed CAR‐T therapy: Clinical spectrum, outcomes, and a framework for neurology–oncology co‐management
Source: Hemasphere. 2026 Jun 15;10(6):e70404. doi: 10.1002/hem3.70404 (PMC13266575; doi:10.1002/hem3.70404)

A

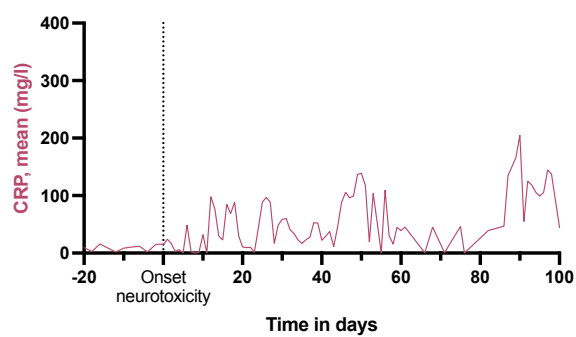

B

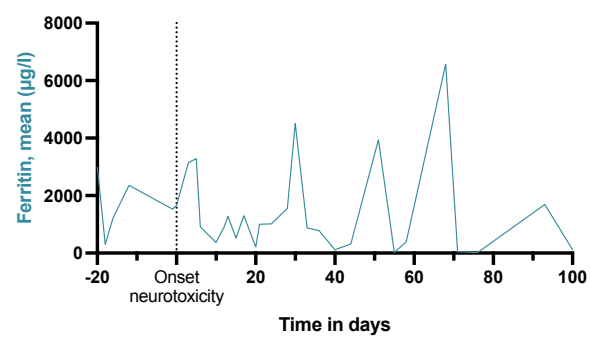

C

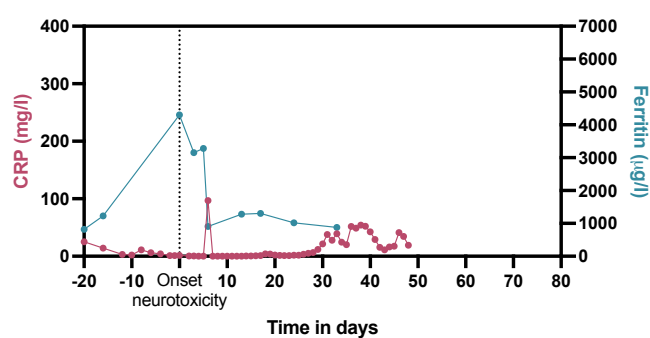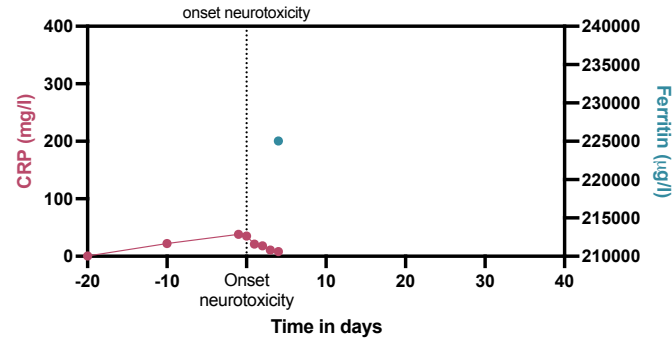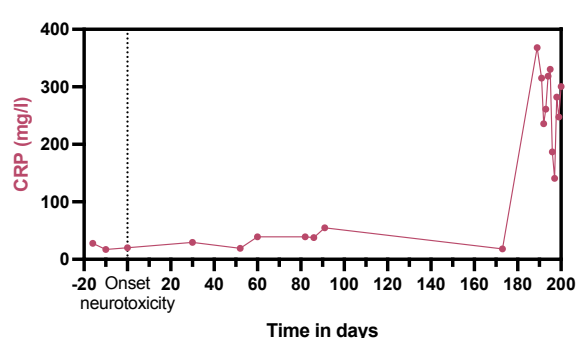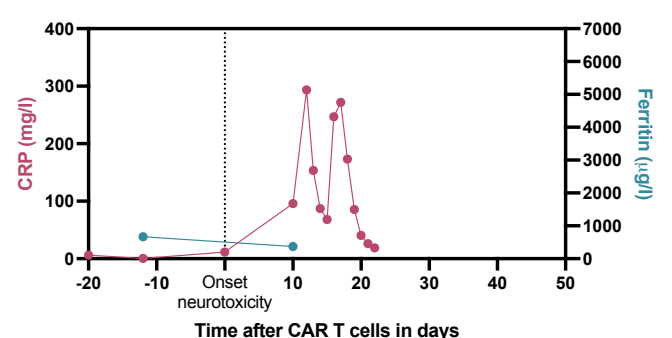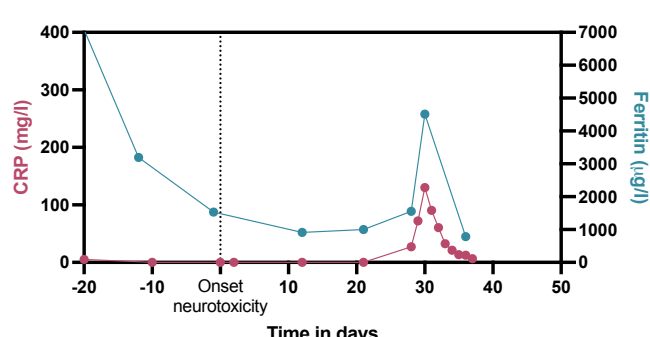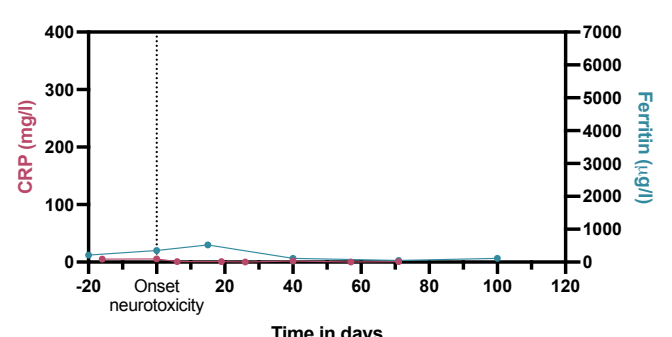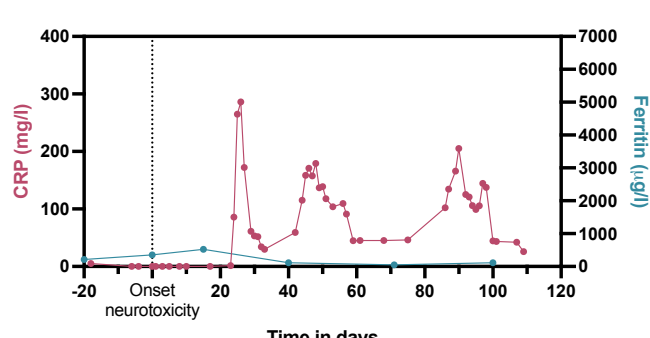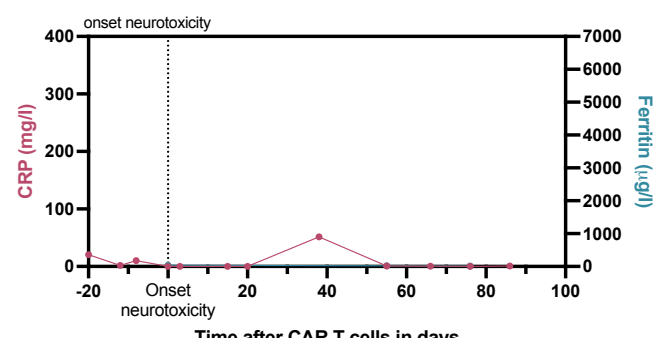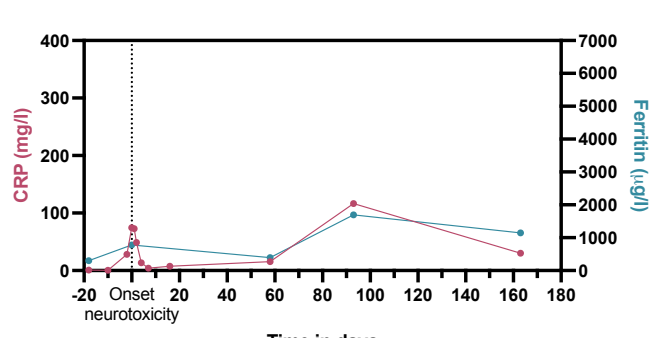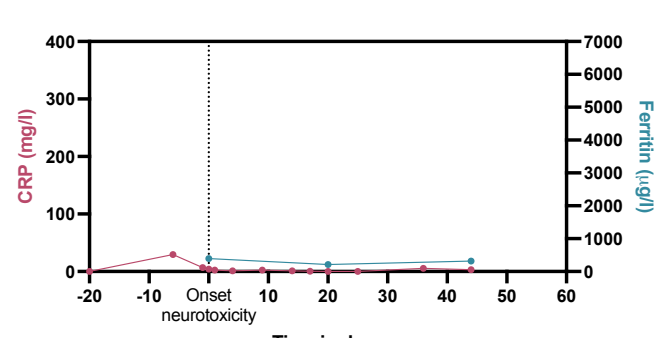

Supplement: Supplementary file 2 — Supporting Information. [file HEM3-10-e70404-s001.pdf]
